# Brain Imaging Analysis Can Identify Participants under Regular Mental Training

**DOI:** 10.1371/journal.pone.0039832

**Published:** 2012-07-03

**Authors:** João R. Sato, Elisa H. Kozasa, Tamara A. Russell, João Radvany, Luiz E. A. M. Mello, Shirley S. Lacerda, Edson Amaro

**Affiliations:** 1 UFABC –Univ. Federal do ABC, Santo André, Brazil; 2 Instituto do Cérebro, Hospital Israelita Albert Einstein, São Paulo, Brazil; 3 Department of Psychobiology – UNIFESP – Univ. Federal De São Paulo, São Paulo, Brazil; 4 King’s College London, Institute of Psychiatry, London, United Kingdom; 5 Department of Physiology, Univ. Federal de São Paulo, São Paulo, Brazil; National Research & Technology Council, Argentina

## Abstract

Multivariate pattern recognition approaches have become a prominent tool in neuroimaging data analysis. These methods enable the classification of groups of participants (e.g. controls and patients) on the basis of subtly different patterns across the whole brain. This study demonstrates that these methods can be used, in combination with automated morphometric analysis of structural MRI, to determine with great accuracy whether a single subject has been engaged in regular mental training or not. The proposed approach allowed us to identify with 94.87% accuracy (p<0.001) if a given participant is a regular meditator (from a sample of 19 regular meditators and 20 non-meditators). Neuroimaging has been a relevant tool for diagnosing neurological and psychiatric impairments. This study may suggest a novel step forward: the emergence of a new field in brain imaging applications, in which participants could be identified based on their mental experience.

## Introduction

Pioneers in neuroscience studied patients with lesions and associated behavioural abnormalities, such as the classic case of Phineas Gage [1], in order to determine aspects of brain function. The advent of neuroimaging provided sufficient detail to enable the detection of brain damage *in vivo*, by the naked eye, and created the basis for neuroradiology [2]. Modern advances in neuroimaging, along with the use of computers, have resulted in more precise automated quantitative analysis. However, subtle differences in images were still difficult to identify accurately, until the application of Machine Learning methods for classification of brain images, such as Support Vector Machine (SVM [3]).

These computational methods of pattern recognition have been used to aid discrimination of clinical brain pathologies associated with easily identifiable behavioural disorders [4], [5]. Indeed, most studies focus on identifying participants with psychiatric or neurological conditions. However, less is known about the ability of these methods to classify the “mental habits” of a non-clinical population based only on information extracted from the brain. For example, suppose clinicians observe a group of subjects on a street market. It may not be too difficult to diagnose a person with autism. However, in the same scene it will be difficult to guess whether a person practices some form of mental training such as meditation.

Previous research has revealed that meditation can be associated with changes in brain function and morphology. For example, Lutz et al. [6] demonstrated that long-term Buddhist meditation practitioners were able to self-induce sustained electroencephalographic high-amplitude gamma-band oscillations and phase-synchrony during meditation. This was particularly apparent at lateral frontoparietal electrodes. Kozasa et al. [7] compared the neural activity of non-meditators and meditators during a task which assessed attention (the Stroop Word-Color Task). Non-meditators showed greater activity than meditators in the right medial frontal, middle temporal, precentral and postcentral gyri and the lentiform nucleus. There were no regions with greater activity in meditators relative to non-meditators. Therefore, non-meditators required greater neural activation compared to regular meditators to achieve equivalent behavioural performance. This supports the hypothesis that meditation training results in greater efficiency via improved sustained attention and impulse control.

In addition, there is evidence that long-term meditation practice is associated with increased cortical thickness. Lazar et al. [8] reported that prefrontal cortex and right anterior insula were thicker in meditators compared to matched controls. These areas are thought to be involved in attention, interoception and sensory processing. Alternatively, Hölzel et al. [9] compared Vipassana meditators with non-meditators and found greater grey matter concentration in the right anterior insula, left inferior temporal gyrus and right hippocampus.

The current study looks to build on this previous research by asking: is it possible to determine whether a person regularly meditates using only their structural brain image? We set out to explore this question by classifying participants by their expertise in meditation and then attempting to identify subtle differences between participants engaged in regular meditation and those who do not meditate. A pattern recognition approach based on SVM and feature selection was applied as a tool for automated classification.

## Materials and Methods

This project was approved by the Ethics Committee of the Instituto Israelita de Ensino e Pesquisa Albert Einstein - Brazil (no. 07/762). Participants taking part in the study were given adequate information before participating and freely signed a consent form.

### Participants

Participants were recruited from mailing lists and were split into regular meditators (19 subjects) or non-meditators (20 subjects) dependent on their responses. Regular meditators were considered to be those who practised meditation three times a week, and had been practising for at least three years. Non-meditators were those who reported practising less than once a week, or not at all.

The groups were matched for age (meditators: 45.47±9.47; non-meditators: 43.80±9.35), gender (meditators: 8M/11F; non-meditators: 9M/11F) and education level (meditators: 78% undergraduate degree, 22% post-graduate; non-meditators: 65% undergraduate degree, 25% post-graduate, 10% secondary school). There was no statistically significant difference between groups on any of these factors. On average, the meditator group had been regularly meditating for 8.5±4.1 years. The styles of meditation used in this group were: “zazen” (N = 4), mantra meditation (N = 2) mindfulness of breathing (N = 6), kriya yoga meditation (N = 4) and meditation associated with hatha yoga (N = 3).

Participants were screened for possible mental health problems, on-going psychological or psychiatric treatment, and use of psychotropic drugs under the supervision of a psychologist and a neuropsychiatrist. In addition, all participants were evaluated on the day of MRI scanning for depression (Beck Depression Inventory [10]), anxiety (Beck Anxiety Inventory [11]), mindfulness (Mindfulness Attention Awareness Scale [12]), and self-compassion (Self-Compassion Scale [13]). There was a significant difference in anxiety levels between the groups, with greater anxiety reported by non-meditators. However, neither group exhibited clinically relevant anxiety (Table S1).

Interviews were conducted *a posteriori* to assess diet and exercise habits. As the interviews took place after the MRI scan, only 13 regular meditators and 15 non-meditators could be contacted. There was no statistically significant difference in diet (vegan, lacto vegetarians, ovo-lacto vegetarians, meat eaters) between the groups (p-value = 0.583, Fisher’s Exact Test). There was also no significant difference in exercise habits (proportion undertaking physical activity at least once a week) between the groups (p-value = 1.000, Fisher’s Exact Test). Furthermore, the groups were similar in the categories of physical activity undertaken by participants (aerobic, anaerobic, stretching, or more than one category of activity; p-value = 0.373, Fisher’s Exact Test). See Table S2 for more detailed information on these possible confounders.

### MRI Acquisition

A high resolution MR image was acquired for each participant using a Siemens 3.0T Magnetom Tim Trio System, We used a MPRAGE T1-weighted sequence (matrix 1×1×1 mm voxel, TR = 2500 ms, TE = 3.45 ms, FOV = 265 mm, inversion time = 1100, flip angle 7 degrees).

### Structural Image Processing

The T1 weighted structural images of all participants were processed using automated cortical and subcortical segmentation (aseg.volume.stats and bilateral aparc.volume.stats files, see Information S1) within the recon-all pipeline of the Freesurfer package (http://surfer.nmr.mgh.harvard.edu). This procedure includes cortical surface modelling, spherical coordinate transformation, nonlinear curvature registration, and automated segmentation of cortical and subcortical structures. The estimated volume of each segmented region obtained using this routine was then used as the input to the classifiers. Further details about recon-all pipeline can be found at [14], [15], [16]. *Note*: Freesurfer software labels the basal putamen as ‘vessel’, since it is an area with prominent vascular space.

### Classification

The pattern recognition method used in this study was the linear two-classes (regular meditators/non-meditators) Support Vector Machine (SVM). To implement this method, we used e1071 package (which provides an interface to the libSVM library [17], www.csie.ntu.edu.tw/~cjlin/libsvm/) within the open source R environment (www.r-project.org). Detailed information about SVM implementation can be found in previous publications [3].

In brief, the basic idea when using pattern recognition analyses is to try to predict the class of an observation (e.g. controls vs patients) based on selected predictor variables (e.g. image features). The two-classes SVM method used in this study is a supervised machine learning approach. This means that the method “learns” to discriminate between two classes based on correctly categorised training data (accurate class and predictor variables from example data). When these classification rules are sufficiently “learnt” then SVM is able to generate class predictions for novel observations (test data). SVM has been used in conjunction with neuroimaging to discriminate patients from controls [18], [19], [20] and also to differentiate distinct brain states based on functional MRI [21], [22]. In the majority of these studies, the predictor variables were specific measures at each voxel of the brain (e.g. gray-matter coefficients, normalized fMRI signal, etc.) and the classes were the disease (present or absent) or experimental condition (e.g.: Task A or Task B in fMRI studies). One of the appealing properties of pattern recognition methods compared to conventional t-tests is that the former is able to generate predictions (and thus assess the amount of predictive information contained within a set of variables), and not only evaluate whether a variable is statistically different between groups.

The current study evaluated whether the information contained in structural (T1 weighted) images was capable of predicting or discriminating between regular meditators and non-meditators. The volumes of each segmented region (121 areas, expressed in cubic millimeters) were used as the variables (features) for group prediction. The names of these predictor variables can be found in Supplementary Information. A feature selection step was included during classification analysis to reduce the influence of irrelevant variables, and also highlight the brain regions containing the most discriminant information. In this way, feature selection can be used as a brain mapping tool.

One of the main dangers when performing classification analysis is double dipping and overfitting, which may lead to unreliable estimates of classifier’s accuracy. These problems can be even worse when a feature selection step is included. In order to avoid these problems, the classifier’s accuracy was estimated based on a first-level leave-one-subject-out procedure and the feature selection was carried out in a second-level nested-leave-one-subject out procedure. This second process was required to guarantee that the information from the specific test subject removed at first-level leave-one-out analysis was only used to estimate prediction accuracy and was not contained within the SVM training data. The feature selection, classification, and accuracy estimation were performed via the following steps:

**Figure 1:**
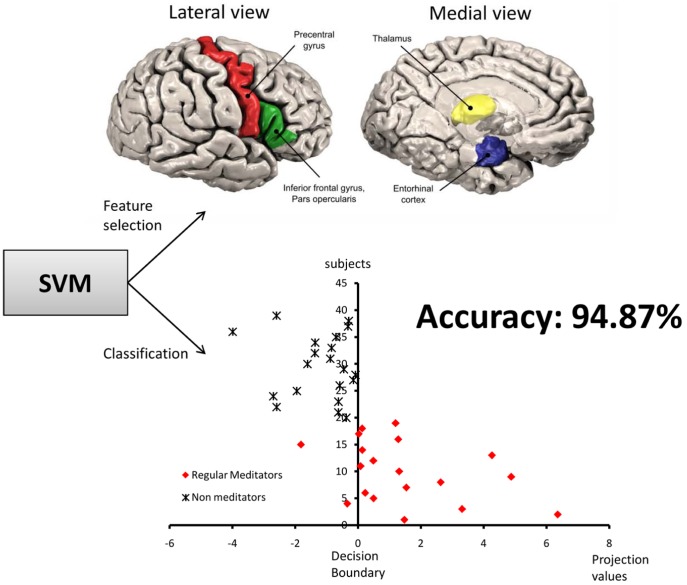
Classification of regular meditators and non-meditators using support vector machines (SVM). Regions identified by the SVM as containing discriminative information used to consistently predict the groups (right precentral gyrus, left entorhinal cortex, right pars opercularis cortex, right basal putamen, and bilateral thalamus). These five regions were selected by SVM in an all leave-one-subject-out iterations, with 94.87% accuracy. The bottom of the figure depicts the projection values of each subject and the decision boundary.

Step 1) Leave one subject out of the sample (first-level leave-one-out);
Step 2) Remove the effects of gender and age from each feature (predictor variable) of the training data by using a multiple linear regression analysis. The corrected training data are the residuals of this regression;
Step 3) Normalize each feature of the corrected training data to have mean zero and variance one. This data is referred as the normalized training data;
Step 4) Leave-one-out implementation:
Step 4.1) Leave another subject out of the normalized training data (second-level leave-one-out);
Step 4.1.a) Train the linear SVM using the respective normalized training data and its label vector (which specifies the groups).
Step 4.1.b) Rank the SVM decision function coefficients (hyperplane coefficients) by their absolute values. This step will provide a rank vector describing the relevance of each feature to the groups’ discrimination;
Step 4.1.c) Feature selection: Remove the most irrelevant feature from the normalized training data;
Step 4.1.d) Train the SVM using the normalized training data obtained in step 4.1.c;
Step 4.1.e) Predict the class of the subject left out in step 4.1;
Step 4.1.f) Return to step 4.1.c and repeat until all features have been removed;
Step 4.2) Return to step 4.1 until all iterations of second-level leave-one-subject-out have been carried out;
Step 5) Compute the classification accuracies of the second-level leave-one-out for different numbers of features. Obtain the number of features, Q, which maximizes the second-level leave-one-out accuracy;
Step 6) Train the linear SVM using the normalized training data obtained in step 3;
Step 7) Obtain the rank vector in the same way as step 4.1.b;
Step 8) Use the rank vector to build a normalized training data consisting solely of the Q (estimated in step 5) most discriminant features;
Step 9) Train the linear SVM using the normalized training data from step 8;
Step 10) Apply the covariate correction and normalization (based on the parameters from step 2 and 3) to the features of the subject left out in step 1 (first-level leave-one-out);
Step 11) Classify the subject left out (first-level) using the test data from step 10;
Step 12) Return to step 1 until all iterations of first-level leave-one-subject-out have been carried out;
Step 13) Compute the first-level leave-one-out accuracy;

**Figure 2:**
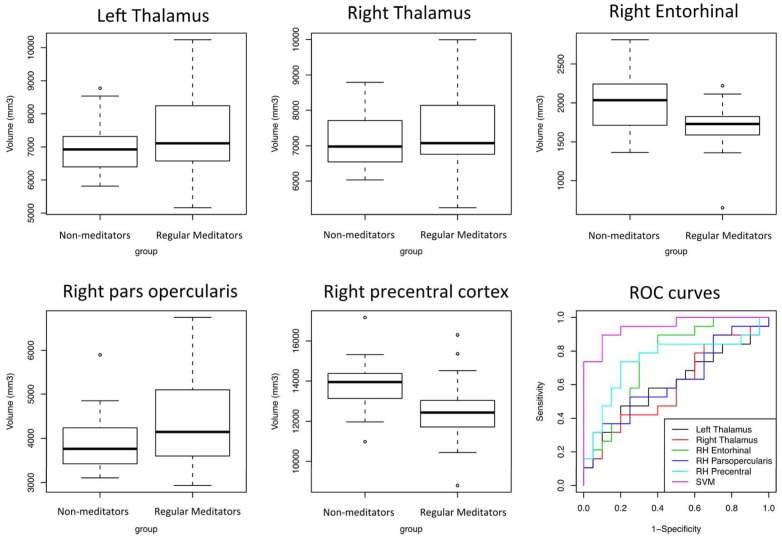
Boxplot illustrating the volumetric information of the regions containing the greatest discriminative information, and ROC curves.

Finally, the p-value for the significance of the first-level leave-one-out accuracy was obtained by using the Binomial distribution. One important point to be mentioned is that the number of features (see step 5) used by SVM is different for each iteration of the first-level leave-one-out. The most discriminant features (brain regions) referred to in the Results and Discussion sections are those which were selected by SVM across all iterations. However, the classification results are based on all the discriminant features found in all leave-one-out iterations.

## Results

It was possible to identify whether a participant belonged to the regular meditator or non-meditator group with 94.87% accuracy (37 participants from 39, p<0.001, accuracy estimated from first-level leave-one-subject-out) using SVM analysis of the volumetric data from several brain regions. The regions containing the most discriminative information, from 121 areas considered, were: right precentral gyrus, left entorhinal cortex, right pars opercularis cortex, right basal putamen, and bilateral thalamus (Figure 1). Boxplots of regional volumes and ROC curves are shown in Figure 2. None of these areas had the same prediction accuracy when employed in isolation, with accurate classification only possible when the spatially distributed areas were used in combination.

## Discussion

Support vector machines seem to be a promising tool for use in disease studies, but we investigated whether this technique could classify healthy participants on the basis of their mental training experience in meditation. Using a combination of neuroimaging and SVM methods, we have shown for the first time that it is possible to classify a particular healthy participant into one of two subgroups, regular meditators and non-meditators, and to identify those brain regions containing the most discriminative information for this classification.

Meditation practice was chosen as the subject of this study because it involves purely mental training, and does not entail the development of strong physical abilities which could act as a potential confound. Physical training has been implicated in changes in brain morphology and function, for example after sports or musical training [23], [24], [25]. In addition, meditation practise has been associated with the development of positive qualities such as emotional control, attention, and a reduction in stress [26]. We investigated a mixed group of regular meditators in order to examine whether practising meditation alters brain morphology to an extent whereby these persons can be accurately classified. If possible, this would suggest that neuroimaging techniques may be able to go beyond helping diagnose brain pathologies, and become a more refined instrument which allows “diagnosis and classification” of differences in “normal” brains.

The areas which contained the greatest discriminative information between regular meditators and non-meditators were sensory and motor-related regions (Figure 1). This finding is in accordance with the ability of meditation to encourage awareness of the sensations entering the brain, selective control over this incoming sensory-motor information and increased internal observation during a period of physical stillness [7], [8].

The results of this study provide a proof-of-concept, demonstrating the ability of pattern analysis techniques and neuroimaging data to discriminate differences in healthy brains dependent on previous experience. Replication of these results in similarly healthy populations would be necessary to confirm these initial results and improve their generalizability. It is possible that the results shown here are influenced by other differences between the regular meditator and non-meditator groups such as educational level, mental health, diet and physical activity. However, of the co-variables recorded, only anxiety levels differed between the groups, with both groups reporting anxiety far below clinical levels. As previously stated the areas of most discrimination were in sensory and motor areas, which makes it unlikely that anxiety had any influence on the results.

It is interesting to hypothesize that, in the future, brain imaging techniques could be applied not only to diagnose disease or injury, but perhaps also to a novel field where persons may be characterised based on their mental experience. We may wonder if it could be possible to identify a more compassionate person, someone who is a natural leader, or even a person who is likely to behave honestly, and speculate about the possible legal implications [27]. Such research may generate interesting information about the effects of mental experience on the brain, but may also raise serious ethical issues. However, the combination of neuroimaging data and SVM methods has the potential to improve prognostic information about how to better assess the long term effects of people’s mental attitudes.

## Supporting Information

Table S1 **Psychological aspects between regular meditators and non-meditators.**
(DOCX)

Table S2 **Distribution of Diet and physical activities of the participants (absolute frequency).**
(DOCX)

Information S1 **Regions from Freesurfer parcellation used as predictor variables.**
(DOCX)
