# Supplementary material for: Brain Imaging Analysis Can Identify Participants under Regular Mental Training
Source: PLoS One. 2012 Jul 3;7(7):e39832. doi: 10.1371/journal.pone.0039832 (PMC3389014; doi:10.1371/journal.pone.0039832)
Supplement: Table S1 — Psychological aspects between regular meditators and non-meditators. (DOCX) [file pone.0039832.s001.docx]

Table S1 - Supplementary material: Psychological aspects between regular meditators and non-meditators

| Psychological measure | Regular meditator | Non-meditator | P* |
| --- | --- | --- | --- |
| Anxiety | 1.37± 1.77 | 3.75±3.31 | 0.008 |
| Depression | 3.79±3.26 | 5.30±3.98 | 0.202 |
| Mindfulness | 71.37±10.85 | 64.65±10.43 | 0.314 |
| Self-compassion | 23.7±3.37 | 22.57±3.33 | 0.056 |

Data presented in Mean ± S.D. / * t test for two independent samples
