# Supplementary material for: Brain Imaging Analysis Can Identify Participants under Regular Mental Training
Source: PLoS One. 2012 Jul 3;7(7):e39832. doi: 10.1371/journal.pone.0039832 (PMC3389014; doi:10.1371/journal.pone.0039832)
Supplement: Table S2 — Distribution of Diet and physical activities of the participants (absolute frequency). (DOCX) [file pone.0039832.s002.docx]

Table S2 - Supplementary material: Distribution of Diet and physical activities of the participants (absolute frequency)

|  | **Group** | **Non-meditators** | **Regular meditators** | **Significance** |
| --- | --- | --- | --- | --- |
| Specific category of diet | Vegan | 1 | 2 | 0.583^F^ |
|  | Lacto/ Ovo-lacto vegetarian | 2 | 3 |  |
|  | Eat meat | 12 | 8 |  |
| Physical activity | Yes | 10 | 8 | 1.000^F^ |
|  | No | 5 | 5 |  |
| Category of physical activity | Aerobic | 7 | 2 | 0.214^F^ |
|  | Streching | 2 | 4 |  |
|  | More than one category of activity | 1 | 2 |  |
| Weekly time of physical activity | <120 min | 2 | 1 | 0.214^F^ |
|  | 121 to 240 min | 6 | 2 |  |
|  | >120 min | 2 | 5 |  |

**Legend:** ^F^ Fisher's Exact Test.
