# Supplementary material for: Brain Imaging Analysis Can Identify Participants under Regular Mental Training
Source: PLoS One. 2012 Jul 3;7(7):e39832. doi: 10.1371/journal.pone.0039832 (PMC3389014; doi:10.1371/journal.pone.0039832)
Supplement: Information S1 — Regions from Freesurfer parcellation used as predictor variables. (DOCX) [file pone.0039832.s003.docx]

**Supplementary Information**

The names of the 121 variables used as input to SVM (obtained from aseg and aparc files from recon-all pipeline of Freesurfer package) are the following:

| Left-Cerebral-White-Matter |
| --- |
| Left-Cerebral-Cortex |
| Left-Lateral-Ventricle |
| Left-Inf-Lat-Vent |
| Left-Cerebellum-White-Matter |
| Left-Cerebellum-Cortex |
| Left-Thalamus-Proper |
| Left-Caudate |
| Left-Putamen |
| Left-Pallidum |
| 3rd-Ventricle |
| 4th-Ventricle |
| Brain-Stem |
| Left-Hippocampus |
| Left-Amygdala |
| CSF |
| Left-Accumbens-area |
| Left-VentralDC |
| Left-vessel |
| Left-choroid-plexus |
| Right-Cerebral-White-Matter |
| Right-Cerebral-Cortex |
| Right-Lateral-Ventricle |
| Right-Inf-Lat-Vent |
| Right-Cerebellum-White-Matter |
| Right-Cerebellum-Cortex |
| Right-Thalamus-Proper |
| Right-Caudate |
| Right-Putamen |
| Right-Pallidum |
| Right-Hippocampus |
| Right-Amygdala |
| Right-Accumbens-area |
| Right-VentralDC |
| Right-vessel |
| Right-choroid-plexus |
| 5th-Ventricle |
| WM-hypointensities |
| Left-WM-hypointensities |
| Right-WM-hypointensities |
| non-WM-hypointensities |
| Left-non-WM-hypointensities |
| Right-non-WM-hypointensities |
| Optic-Chiasm |
| CC_Posterior |
| CC_Mid_Posterior |
| CC_Central |
| CC_Mid_Anterior |
| CC_Anterior |
| BrainSegVol |
| IntraCranialVol |
| lh_superiorparietal_volume |
| lh_caudalanteriorcingulate_volume |
| lh_cuneus_volume |
| lh_lingual_volume |
| lh_isthmuscingulate_volume |
| lh_rostralanteriorcingulate_volume |
| lh_middletemporal_volume |
| lh_transversetemporal_volume |
| lh_caudalmiddlefrontal_volume |
| lh_fusiform_volume |
| lh_parstriangularis_volume |
| lh_temporalpole_volume |
| lh_unknown_volume |
| lh_postcentral_volume |
| lh_superiortemporal_volume |
| lh_pericalcarine_volume |
| lh_entorhinal_volume |
| lh_rostralmiddlefrontal_volume |
| lh_parsorbitalis_volume |
| lh_inferiortemporal_volume |
| lh_frontalpole_volume |
| lh_posteriorcingulate_volume |
| lh_medialorbitofrontal_volume |
| lh_lateraloccipital_volume |
| lh_bankssts_volume |
| lh_paracentral_volume |
| lh_insula_volume |
| lh_supramarginal_volume |
| lh_parahippocampal_volume |
| lh_parsopercularis_volume |
| lh_inferiorparietal_volume |
| lh_precentral_volume |
| lh_superiorfrontal_volume |
| lh_lateralorbitofrontal_volume |
| lh_precuneus_volume |
| rh_superiorparietal_volume |
| rh_caudalanteriorcingulate_volume |
| rh_cuneus_volume |
| rh_lingual_volume |
| rh_isthmuscingulate_volume |
| rh_rostralanteriorcingulate_volume |
| rh_middletemporal_volume |
| rh_transversetemporal_volume |
| rh_caudalmiddlefrontal_volume |
| rh_fusiform_volume |
| rh_parstriangularis_volume |
| rh_temporalpole_volume |
| rh_unknown_volume |
| rh_postcentral_volume |
| rh_superiortemporal_volume |
| rh_pericalcarine_volume |
| rh_entorhinal_volume |
| rh_rostralmiddlefrontal_volume |
| rh_parsorbitalis_volume |
| rh_inferiortemporal_volume |
| rh_frontalpole_volume |
| rh_posteriorcingulate_volume |
| rh_medialorbitofrontal_volume |
| rh_lateraloccipital_volume |
| rh_bankssts_volume |
| rh_paracentral_volume |
| rh_insula_volume |
| rh_supramarginal_volume |
| rh_parahippocampal_volume |
| rh_parsopercularis_volume |
| rh_inferiorparietal_volume |
| rh_precentral_volume |
| rh_superiorfrontal_volume |
| rh_lateralorbitofrontal_volume |
| rh_precuneus_volume |
